# Phase 2 multicentre trial investigating intermittent and continuous dosing schedules of the poly(ADP-ribose) polymerase inhibitor rucaparib in germline BRCA mutation carriers with advanced ovarian and breast cancer

**DOI:** 10.1038/bjc.2016.41

**Published:** 2016-03-22

**Authors:** Yvette Drew, Jonathan Ledermann, Geoff Hall, Daniel Rea, Ros Glasspool, Martin Highley, Gordon Jayson, Julieann Sludden, James Murray, David Jamieson, Sarah Halford, Gary Acton, Zoe Backholer, Raffaella Mangano, Alan Boddy, Nicola Curtin, Ruth Plummer

**Affiliations:** 1 Northern Institute for Cancer Research and the Northern Centre for Cancer Care, Newcastle Freeman Hospital Newcastle, Newcastle 0191 2139386, UK; 2 UCL Cancer Institute, CR-UK and UCL Cancer Trials Centre, 90 Tottenham Court Road, London W1T 4TJ, UK; 3 St James's University Hospital, Cancer Research UK Clinical Cancer Centre in Leeds, Beckett Street, Leeds LS9 7TF, UK; 4 University of Birmingham, School of Cancer Sciences, Edgbaston, Birmingham B15 2TT, UK; 5 Beatson West of Scotland Cancer Centre, Medical Oncology 1053 Great Western Road, Glasgow G12 0YN, UK; 6 Derriford Hospital Plymouth Oncology Centre, Plymouth, UK; 7 University of Manchester, Paterson Institute for Cancer Research, Translational Angiogenesis Group, Wilmslow Road, Manchester, M20 4BX, UK; 8 Northern Institute for Cancer Research, Newcastle University, Framlington Place, Newcastle, UK; 9 Cancer Research UK, Drug Development Office, The Angel Building, 407 St John Street, London EC1V 4AD, UK; 10 Cancer Research UK, Centre for Drug Development Drug Development, Angel Building, London EC1V 4AD, UK

## Abstract

**Background:**

Rucaparib is an orally available potent selective small-molecule inhibitor of poly(ADP-ribose) polymerase (PARP) 1 and 2. Rucaparib induces synthetic lethality in cancer cells defective in the homologous recombination repair pathway including BRCA-1/2. We investigated the efficacy and safety of single-agent rucaparib in germline (g) BRCA mutation carriers with advanced breast and ovarian cancers.

**Methods:**

Phase II, open-label, multicentre trial of rucaparib in proven BRCA-1/2 mutation carriers with advanced breast and or ovarian cancer, WHO PS 0–1 and normal organ function. Intravenous (i.v.) and subsequently oral rucaparib were assessed, using a range of dosing schedules, to determine the safety, tolerability, dose-limiting toxic effects and pharmacodynamic (PD) and pharmacokinetic (PK) profiles.

**Results:**

Rucaparib was well tolerated in patients up to doses of 480 mg per day and is a potent inhibitor of PARP, with sustained inhibition ⩾24 h after single doses. The i.v. rucaparib (intermittent dosing schedule) resulted in an objective response rate (ORR) of only 2% but with 41% (18 out of 44) patients achieved stable disease for ⩾12 weeks and 3 patients maintaining disease stabilisation for >52 weeks. The ORR for oral rucaparib (across all six dose levels) was 15%. In the oral cohorts, 81% (22 out of 27) of the patients had ovarian cancer and 12 out of 13, who were dosed continuously, achieved RECIST complete response/partial response (CR/PR) or stable disease (SD) ⩾12 weeks, with a median duration of response of 179 days (range 84–567 days).

**Conclusions:**

Rucaparib is well tolerated and results in high levels of PARP inhibition in surrogate tissues even at the lowest dose levels. Rucaparib is active in gBRCA-mutant ovarian cancer and this activity correlates with platinum-free interval. The key lessons learned from this study is that continuous rucaparib dosing is required for optimal response, the recommended phase 2 dose (RP2D) for continuous oral scheduling has not been established and requires further exploration and, thirdly, the use of a PD biomarker to evaluate dose–response has its limitations.

Poly(ADP-ribose) polymerase (PARP) inhibitors are an exciting development in anticancer therapy (Sonnenblick *et al*, 2015). The superfamily of PARP enzymes consists of 17 members, with PARPs 1–3 being activated by, and promoting the repair of, DNA breaks (Schreiber *et al*, 2006). The most abundant PARPs, PARP-1 and 2, play an essential role in the repair of DNA single-strand breaks (SSBs) via the base excision repair/single-strand break repair (BER/SSBR) pathway. Poly(ADP-ribose) polymerase inhibition results in accumulation of unrepaired SSBs, leading to collapsed replication forks and DNA double-strand breaks (DSBs). The Homologous Recombination repair (HRR) pathway, in which BRCA1 and BRCA2 are key elements, is essential to the efficient and error-free repair of such lesions (Helleday *et al*, 2007). Germline (g) mutations in either the *BRCA1* or *BRCA2* genes render individuals at high life-time risk of breast and ovarian cancer (Gudmundsdottir and Ashworth, 2006) and these subsequent cancers may have HRR deficiency (HRD). PARP inhibitors (PARPis) have been shown to selectively kill cells and xenografts with HRD by a process known as ‘synthetic lethality' (Bryant *et al*, 2005; Farmer *et al*, 2005). ‘Synthetic lethality' is the concept by which death results from the inactivation of two genes or pathways when inactivation of either gene or pathway alone is nonlethal (Kaelin, 2005). Subsequent early-phase clinical trials of PARPis have shown promising antitumour activity in *BRCA*-mutant cancers with acceptable toxicity profiles (Fong *et al*, 2009; Audeh *et al*, 2010; Tutt *et al*, 2010; Sandhu *et al*, 2013). In addition, PARPis, as single agents, may have a broader application in the treatment of cancers with HRD not directly due to gBRCA mutations. For example, ∼50% of high-grade serous ovarian cancers (HGSOCs) were shown in The Cancer Genome Atlas Network (TCGAN) molecular analysis to harbour HRD (The Cancer Genome Atlas Research Network, 2011). This HRD included somatic *BRCA* mutations (6–8%), and epigenetic silencing in genes not associated with *BRCA* but essential to HRR function, such as *ATM*, *CHEK2*, *RAD51* and *MRE11A*. Similarly 55% of unselected HGSOCs were found to have HRD using a functional assay of HRR, and be sensitive to PARP inhibition (Mukhopadhyay *et al*, 2010). Induction of HRD in cancers by altering the tumour microenvironment through hypoxia (Chan *et al*, 2010) or by combining PARPis with agents that might downregulate HRR, such as VEGF inhibitors (Lui *et al*, 2014), might render HRR-competent cells sensitive to PARP inhibition. This concept, known as ‘contextual' synthetic lethality, could broaden the application of this class of drugs in the treatment of cancer and is the rationale behind other ongoing clinical trials (www.clinicaltrials.gov).

Rucaparib (CO-338; formally known as PF-01367338 and AG-014699) is a potent selective small-molecule inhibitor of both PARP-1 and PARP-2, with a respective Ki of 0.8 and 0.5 nM (Thomas *et al*, 2007). In addition, it has recently been shown to have activity against the tankyrases TANK1 and 2 otherwise known as PARP5A and PARP5B (Wahlberg *et al*, 2012). The free base compound AG-014447 (8-Fluoro-2-(4-methylaminomethyl-phenyl)-1,3,4,5- tetrahydro-azepino[5,4,3-cd]indol-6-1) is available in phosphate and camphorsulphonic acid salt forms. The phosphate salt (intravenous (i.v.) formulation) was named rucaparib by Clovis Oncology Inc. (Boulder, CO, USA), following their acquisition of the full rights to the agent in 2011. The oral camphorsulphonic acid salt formulation is known as rucaparib camsylate. For the purpose of this report the investigational agent will be termed i.v. and oral rucaparib. In preclinical models AG014699 (i.v. rucaparib compound) inhibits tumour growth in not only mutant *BRCA*1/2 models, but also in those with non-*BRCA* mutant-deficient HR, such as deficient XRCC3 and epigenetically silenced *BRCA*1 (Drew *et al*, 2011a). Phase 1 (in advanced solid tumours) and 2 (in melanoma) studies of i.v. rucaparib in combination with the oral DNA methylating agent temozolomide were completed in 2005 and 2008 respectively (Plummer *et al*, 2008, 2013). No rucaparib-related serious adverse effects were reported in the phase I trial and PARP inhibition of >90% in tumours and surrogate tissues following single i.v. doses were observed.

This is the first study to investigate the antitumour effects of single-agent i.v. and oral rucaparib in patients with gBRCA mutant advanced breast and ovarian cancers. Employing a range of dosing schedules, the safety, tolerability, dose-limiting toxic effects and pharmacodynamic (PD) and pharmacokinetic (PK) profiles of rucaparib are assessed.

## Materials and methods

### Trial design and patient recruitment

**Figure 1:**
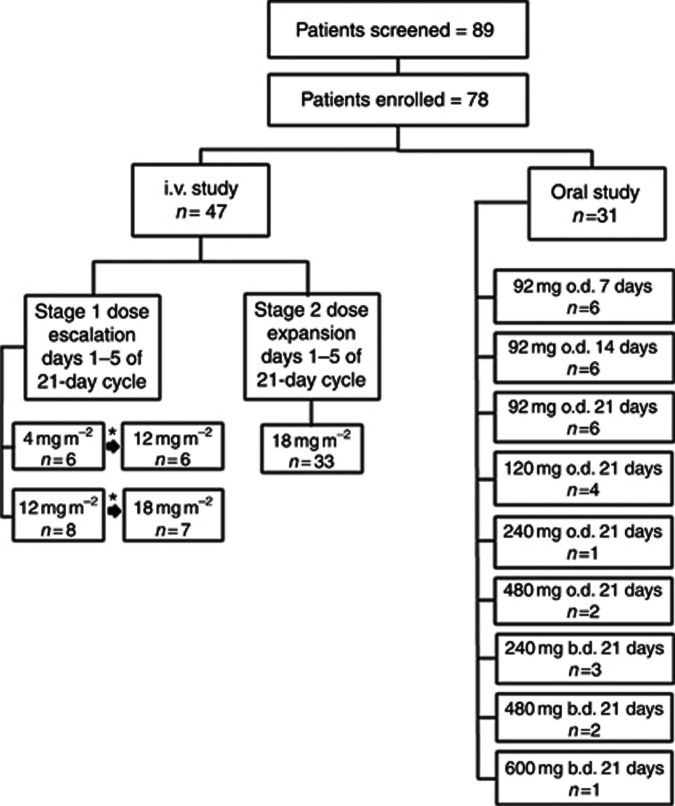
**Phase 2 study schema showing total number of patients screened, subsequently enrolled into the study and treated at each dose level and schedule for both i.v. and oral rucaparib.** The symbol ‘*' represents the same patients who dose escalated in the absence of any dose-limiting toxicity.

The trial was originally designed as an open-label, multicentre, phase II study of i.v. rucaparib, given as a 30-min infusion daily for 5 days of each 21-day cycle in patients with proven gBRCA-1/2 mutations and locally advanced or metastatic breast cancer, or advanced ovarian cancer. At the time of study conception (2006), there were no published clinical safety data about the use of PARPis in gBRCA mutation carriers. It was also unknown whether differences in response would be observed between the *BRCA1* and *BRCA2* mutation carriers or the breast and ovarian cancers. The study therefore comprised a short dose-escalation phase (stage 1), using cohorts of *n*=6 (3 *BRCA1* and 3 *BRCA2*) at each dose level, as outlined in Figure 1, and a proof of principle phase (stage 2) using the recommended safe i.v. dose established in stage 1. In stage 2, patients were stratified into four groups to assess response based on gBRCA mutation status (1 or 2) and tumour type (breast or ovary). In stage 1, after recruitment to the initial cohort, patients were recruited to subsequent cohorts provided there were no dose-limiting toxicities (DLTs) at the previous dose level.

Preliminary analysis of the PD and clinical response data in the first 38 patients treated suggested that a more continuous schedule was required for efficacy (Drew *et al*, 2011b) Therefore, when in 2011 an oral, tablet formulation of rucaparib (rucaparib camsylate) became available through Clovis Oncology, recruitment to the i.v. cohorts was suspended and the study design amended. The study reopened (on 25 October 2011) to investigate oral rucaparib at higher doses and more prolonged schedules, including continuous dosing. Patients who remained on study at the time of the switch to oral dosing continued with i.v. dosing until study withdrawal. The oral rucaparib starting dose was set at 92 mg daily that is bioequivalent to an 18 mg m^−2^ i.v. dose using the calculation: oral dose=(i.v. dose × body surface area)/oral bioavailability. Body surface area (BSA) was set at 1.75 m^2^ (median BSA of the first 42 patients treated within the i.v. study). At this time, bioavailability was estimated at 34% (data on file at Clovis); however, subsequent studies have shown it to be 38% and dose independent (Molife *et al*, 2013).

The oral study initially investigated increasing duration of dosing (7, 14 and 21 days) at the set dose of 92 mg once daily (o.d.) within a 21-day cycle. Higher dose levels and more frequent dosing (twice daily (b.d.)) were then investigated in order to determine the optimal dose and regimen of oral rucaparib. Following the reporting of phase 2 clinical data suggesting that non-gBRCA HGSOCs respond to PARPis (Gelmon *et al*, 2011), recruitment to oral rucaparib was also opened to patients with HGSOC with unknown gBRCA mutation status.

The study was conducted in accordance with the Good Clinical Practice guidelines and the Declaration of Helsinki. The protocol and subsequent amendments were approved by the MHRA, a multicentre research ethics committee, local Research and Development departments and Cancer Research UK (the sponsor). Patients were enrolled and treated at seven centres across the United Kingdom.

All patients provided written informed consent. Inclusion criteria included histologically documented malignancy, locally advanced or metastatic breast cancer or advanced ovarian cancer (including epithelial, fallopian tube and primary peritoneal cancer), evidence of gBRCA1/2 mutation, WHO performance status (PS) 0 or 1, age ⩾18 years, life expectancy ⩾12 weeks, no more than 5 lines of previous chemotherapy, adequate bone marrow, liver and renal function and measurable disease as defined by the Response Evaluation Criteria in Solid Tumours (RECIST version 1.0) (Therasse *et al*, 2000). For ovarian cancer patients, >2 months or ⩾6 months must have elapsed since the last platinum-containing regimen for the *BRCA* mutant and the HGSOC patients respectively. Patients were excluded if they had received prior PARPi treatment, had brain metastases or significant comorbidities. Patients were treated until disease progression or study withdrawal for other reasons.

### Study end points

The primary end points were to determine the tumour objective response rate (ORR) and the toxicity of i.v. and oral rucaparib in the study population. Secondary end points were: to determine a tolerable and effective dosing regimen for oral rucaparib to recommend for future studies, time to progression and overall survival, to assess PK and to evaluate the effect of rucaparib on PARP enzyme activity in peripheral blood lymphocytes (PBLs) through a validated PD assay.

Disease response was assessed according to RECIST every 2 cycles (6 weeks). Patients who completed at least 2 cycles at ⩾80% of dose were eligible for response evaluation. Toxicity and tolerability of rucaparib was determined by adverse event monitoring using the Common Terminology Criteria for Adverse Events (CTCAE) version 3.0 (http://ctep.cancer.gov, publish date 9 August 2006). Dose-limiting toxicity was defined as a drug-related adverse event defined by CTCAE version 3.0 occurring in the first and second cycle for i.v. patients in stage 1 and in cycle 1 for all other patients.

Response rates are reported as RECIST complete response and partial response (CR/PR) and RECIST stable disease (SD) for ⩾12 weeks, which is at least 4 treatment cycles.

### PARP activity pharmacodynamics

The PARP enzyme activity levels in surrogate cells (PBLs) were assessed at baseline during cycle 1 on day 1 (D1) pre-dose and in response to rucaparib at the following time-points: end-of -infusion, 4 h post dose and on D2 pre-dose (∼24 h post D1 dose). Patients in the i.v. cohorts who dose escalated had samples taken in cycles 1 and 2. In the oral cohorts, PARP activity was assessed at baseline, cycle 1 D1 pre-dose and following rucaparib at 30 min post dose, 4 h post dose and D2 pre-dose and on D8, D15 and D22 pre-dose (where appropriate). Samples were analysed using a validated assay that uses quantitative immunologic detection of *ex vivo* poly(ADP-ribose) formation (Plummer *et al*, 2005). During the validation of the assay a large interassay variability (40%) was observed between blots (unpublished data, Dr Chris Jones, Newcastle University, Newcastle upon Tyne, UK), making it questionable to compare PARP activity directly between blots using the luminescence values. To combat this and standardise results between assays, the following serially diluted standards of PAR 25, 5, 1, 0.2, 0.04 and 0 pmol ADP-ribose monomer are loaded onto the immunoblot at the same time as the patient cell samples. In addition, all samples for each individual patient were run on the same blot in order to assess changes in PARP activity accurately over time following rucaparib.

### Rucaparib pharmacokinetics

For the i.v. cohorts, plasma samples for PK analysis of rucaparib were taken during cycle 1 on D1 (pre-dose, end of infusion, 1–3 h post dose), D2 pre-dose, D4 (pre-dose, end of infusion, 1–3 h post dose), D5 pre-dose and D5 end of infusion (patients who dose escalated in the i.v. cohorts had samples taken in cycles 1 and 2). In the oral patients, PK samples were taken in cycles 1 and 2 at D1 (pre-dose, 30 min and1/1.5/2.5/4/6 h post dose), D2 pre-dose, D7 (pre-dose, 30 min and 1/1.5/2.5/4/6 h post dose), D8 pre-dose and D15 pre-dose for cohort 1. Cohort 2 (14-day dosing) D7 PKs were done on D14 and in cohort 3 (21 day dosing) and subsequent continuous dosing cohorts on D21. Analysis of rucaparib in plasma samples was performed according to a validated assay (NICR Standard Operating Procedure (SOP) 240) using LC/MS/MS. Rucaparib PK parameters were calculated by non-compartmental analysis with a linear/log-trapezoidal model for AUC using WinNonlin Professional software (Version 5.3, Pharsight, Mountain View, CA, USA).

### Dose-limiting toxicity

Defined as grade 4 neutropenia ⩾5 days, fever ⩾38.5 °C and/or documented infection associated with ⩾grade 3 neutropenia, grade 4 thrombocytopenia ⩾5 days, ⩾grade 3 non-haematological toxicity (excluding nausea, vomiting and diarrhoea if optimal treatments had not been received) and death.

### *BRCA* mutation testing

Only patients with proven gBRCA mutations were eligible for the i.v. study. However, patients who had a strong family history of breast and/or ovarian cancer and were considered by clinical geneticists to be likely carriers of a *BRCA* mutation (Manchester criteria score ⩾20) could give their consent to enrol into the study and undergo *BRCA* mutational analysis (carried out by Myriad Genetics Inc. (Salt Lake City, UT, USA) via UK partner Lab21 Limited, Cambridge, UK) to confirm eligibility before receiving treatment. In the oral dosing cohorts, HGSOC patients with unknown *BRCA* status were permitted to enrol into the study, but underwent *BRCA* testing while on study.

### Data analysis

The study was designed to assess effects of rucaparib in each of the four subgroups defined by *BRCA* mutation 1 or 2 and tumour type breast or ovary. All patients who received at least one dose of study drug were evaluable for toxicity assessment. Only patients who received rucaparib for least 80% of prescribed dose for ⩾2 cycles were evaluable for tumour response. Radiological responses were confirmed by a second assessment performed within 6 weeks of the initial report.

## Results

### Patient demographics and treatment

Patients were recruited to the i.v. cohorts between January 2008 and September 2011 and the oral study between October 2011 and August 2013.

A total of 89 patients consented to the study and were screened for eligibility. Of these, 78 patients (47 in the i.v. phase and 31 in the oral phase) were enrolled into the study. The median age at enrolment was 52 years (range 23–72 years). In all, 48 patients had a *BRCA1* mutation (15 with breast cancer, 33 with ovarian cancer) and 26 had a mutation within *BRCA2* (12 breast, 14 ovary). In addition, four patients with HGSOC and unknown *BRCA* were enrolled in the oral part of the study. Out of these 4 patients, three later underwent *BRCA1/2* mutation genotyping but no mutations in the *BRCA* genes were detected. The remaining patient was not tested. The median number of prior chemotherapy regimens for all patients was 2 (range 1–6) and all of the ovarian cancer patients had received prior platinum-based chemotherapy. Of all the patients, 56% were WHO PS zero.

All 78 patients received at least one dose of the PARPi and were eligible for toxicity assessment. A total of 71 patients completed at least 2 cycles at ⩾80% of dose and were eligible for response evaluation. The study schema with the number of patients treated at each dose level and schedule in both the i.v. and oral dosing cohorts is illustrated in Figure 1.

### Clinical response

The i.v. rucaparib on an intermittent dosing schedule resulted in an ORR of only 2% but 41% (18 out of 44) of patients achieved SD for ⩾12 weeks, with 3 patients maintaining disease stabilisation for >52 weeks (see Table 1). The low ORR combined with PD data showing recovery of PARP enzyme activity during nontreatment days (discussed below) suggested a suboptimal dosing schedule and supported the amendment of the study to investigate the efficacy of continuous oral dosing to prolong PARP inhibition.

The ORR for all oral rucaparib (across all six dose levels and schedules) was 15%, but many patients achieved disease stabilisation, with an overall RECIST SD ⩾12 weeks of 63%. For all dose levels in the continuous oral dosing cohorts (*n*=17), the SD ⩾12 weeks rate was 59% (10 out of 17), with ORR of 18%, as shown in Table 1. In the oral cohorts, 81% (22 out of 27) of the patients had ovarian cancer and 12 out of 13, who were dosed continuously, achieved RECIST CR/PR or SD ⩾12 weeks, with a median duration of response of 179 days (range 84–567 days).

Of the 78 patients treated in the study, 51 had ovarian cancer and all had received prior platinum- based chemotherapy. In this group a higher benefit based on RECIST CR/PR or SD ⩾12 weeks was seen in patients with the longest platinum-free interval (PFI): 81% *vs* 60% *vs* 29% for PFI of >12 months, 6–12 months and <6 months, respectively.

Interestingly, there were no responders by ORR to rucaparib in the breast cancer patients. Of the evaluable patients, 39% (9 out of 23) did achieve SD ⩾12 weeks as best response.

**Figure 2:**
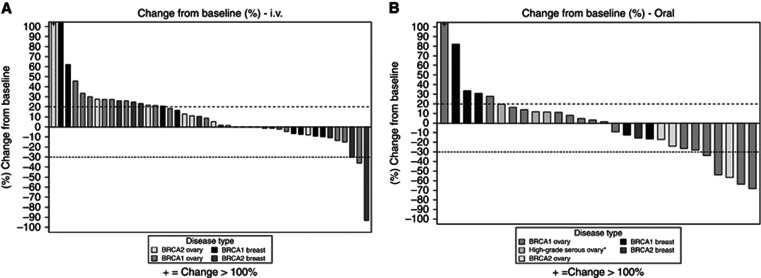
Waterfall plots showing the best percentage change in tumour size over baseline as measured according to RECIST for each assessable patient in (**A**) i.v. cohorts and (**B**) oral cohorts. *High-grade serous ovarian cancer and unknown or wild-type *BRCA*.

Best percentage change in tumour size over baseline for the i.v. cohorts and the oral schedules are shown in the waterfall plots in Figure 2. Both *BRCA1* and *BRCA2* mutation carriers responded to rucaparib. However, because of overall low ORR, subgroup analysis of response by tumour type and BRCA status was not statistically feasible. The ORR results stratified by tumour type and BRCA mutation are shown in Table 1; of note, no objective responses were seen in any of the breast cancer patients for both i.v. and oral rucaparib. Of the four patients with non-gBRCA HGSOC, three were evaluable for response. One patient progressed after 2 cycles and the other two patients had SD for 4 and 8 cycles respectively.

### Toxicity

Rucaparib was well tolerated by patients up to a dose of 480 mg per day. Doses above this level (480 mg b.d. and 600 mg b.d.) resulted in DLTs (CTCAE grade 3 fatigue) and drug discontinuation because of persistent fatigue in 2 patients. No DLTs were seen in the i.v. phase of the study. Table 2 lists the most frequently occurring adverse events (AEs) that were mostly grade 1/2 in severity and manageable with standard therapeutic interventions. The most common treatment related AEs were fatigue (51% all grades) and nausea (36% all grades). There were no reported grade 4/5 AEs and there were no treatment-related deaths on study.

### Pharmacodynamics PARP activity in response to rucaparib

**Figure 3:**
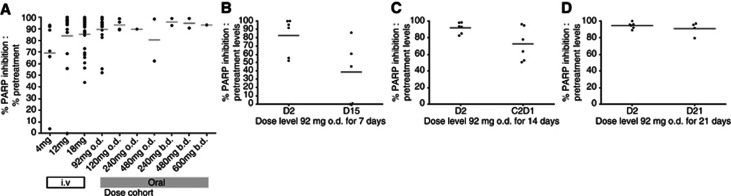
**Scatter plots of data showing % PARP enzyme inhibition levels relative to pretreatment levels (black circles) in patients' PBLs following treatment with rucaparib.** (**A**) All dose levels at ∼24 h following last dose of rucaparib except for the twice daily schedules when levels were taken ∼12 h after last dose. Data for the 92 mg o.d. cohorts only are shown in (**B**) 7 days continuous dosing, (**C**) 14 days continuous dosing and (**D**) 21 days continuous dosing. The line represents the mean % inhibition.

Inhibition of PARP enzyme activity following treatment with rucaparib was evaluated in 71 of the 78 study patients. The i.v. rucaparib is a potent inhibitor of PARP with the mean % inhibition at end of infusion of 98.8%, 95.5% and 88.4% for the 4, 12 and 18 mg m^−2^ dose levels, respectively. At ⩾24 h after a single dose of rucaparib (D2 pre-dose sample time-point), >50% of PARP inhibition was maintained even at the lowest dose level of 4 mg m^−2^ (see Figure 3A).

A total of 31 patients received oral rucaparib at the dose levels shown in Figure 1. Results of the effects of oral rucaparib on PARP-1 enzyme activity are seen in Figure 3, with potent inhibition of PARP, ⩾90% mean inhibition over pre-treatment values at 24 h, after a single dose at all dose levels (except the 480 mg o.d. cohort). In all, 18 patients received 92 mg o.d. dosing: 6 patients for 7 days, 6 patients for 14 days and 6 were dosed continuously for the 21-day cycle at this dose level. This enabled the effects of a more prolonged schedule of the same dose to be assessed in terms of duration of PARP enzyme inhibition and safety. Mean pre-dose PARP activity levels for the three cohorts are shown in Figure 3B. By D15 in the 7-day schedule, clear recovery of PARP enzyme activity was seen. In fact, PARP activity levels higher than the recorded pre-treatment values were observed (mean=195%). After 14 days of dosing, some recovery at cycle 2 day 1 was still observed (mean PARP activity=27% compared with 8% at C2D1). However, at D21 in the 21-day continuous dosing schedule, PARP activity remains clearly suppressed with mean pre-dose levels of only 9%. These data support the use of a continuous dosing schedule of rucaparib to maintain PARP inhibition, minimise recovery of enzyme activity between doses and improve clinical efficacy. Following the three schedules of 92 mg o.d., patients were recruited to cohorts of increasing daily doses of continuous oral rucaparib. In these 13 patients, increasing the continuous oral rucaparib dose >92 mg o.d., or dosing twice daily, did not result in a significantly greater mean PARP enzyme inhibition at 24 h post dose.

### Pharmacokinetics

**Figure 4:**
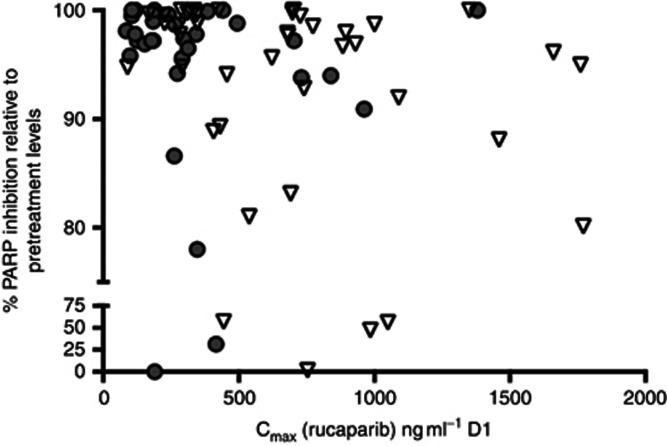
**The PK–PD relationship.** The data show maximum % PARP inhibition levels relative to pretreatment values (grey circles) plotted against C_max_ rucaparib levels (ng ml^−1^) (inverted white triangles) on day 1 of treatment for all i.v. and oral dose cohorts.

The data from patients receiving i.v. rucaparib as a single agent in the current study were largely in line with those reported previously (Plummer *et al*, 2008), with day 1 end-of-infusion concentrations of 246±103, 676±271 and 755±420 ng ml^−1^ at 4, 12 and 18 mg m^−2^, respectively (see Supplementary Data). At the comparator dose level of 92 mg, AUC values for rucaparib after oral dosing were similar to those seen after administration of 18 mg m^−2^ i.v., indicating that bioavailability was ∼33%, similar to that predicted from data of previous bioavailability studies (provided by Clovis). Plasma concentrations and AUC values increased as doses were escalated, as indicated in the Supplementary Table 1, that summarises the main PK parameters at each oral dose level. There was however not a clear relationship between dose and C_max_ or dose and AUC because of the high degree of interpatient variability. The half-life of rucaparib after oral dosing was 9.1±2.7 h. In all but 4 patients, across all doses and for both oral and i.v. administration, >75% PARP inhibition was observed. Regardless of route of administration, there was no discernible PK–PD relationship, with near maximal PARP enzyme inhibition observed even at doses that produced only modest C_max_ values. In addition, although intersubject variation in PK was significant, this did not result in a wide variation of this surrogate PD end point (see Figure 4).

## Discussion

This trial was designed to investigate the efficacy and toxicity of the PARPi rucaparib as a single agent in patients with gBRCA-mutated advanced breast or ovarian cancer. The results show that rucaparib is a well-tolerated potent inhibitor of PARP with >75% PARP inhibition seen in the surrogate PBLs of every patient following rucaparib and sustained inhibition ⩾24 h after single doses, confirming proof of mechanism. Following rucaparib, many patients achieved SD with the continuous oral dosing schedules; however, in the overall study ORR was lower than expected. The reasons are likely to be multifactorial but the intermittent scheduling and the cautious dose escalation were thought to be mainly responsible. The dosing schedule of 5 days out of every 21 days allowed recovery of PARP enzyme activity and consequently loss of synthetic lethality. This is consistent with the data from the oral cohort where 18 patients received rucaparib 92 mg once daily but for different durations within the 21-day cycle. The oral cohorts demonstrate that sustained PARP inhibition (>90%) was observed only with continuous dosing, either daily or twice daily (see Figure 4). In addition, subsequent preclinical studies of rucaparib have demonstrated that a daily dosing schedule of 5 days every 21 days does not result in delay of *BRCA* mutant tumour growth, whereas continuous dosing does (Murray *et al*, 2014). The lack of ORR seen in the breast patients may be less about the schedule and more related to differences in tumour biology in *BRCA* mutant breast cancer as has been reflected in results of other studies where responses have been seen on *BRCA-*mutant ovarian but not breast tumours Gelmon *et al*, 2011).

All of the 51 ovarian cancer patients treated in the study had received prior platinum and it is known in preclinical studies that exposure to platinum agents can result in genetic reversions that result in resistance to PARP inhibitors (Swisher *et al*, 2008). It is not known whether this phenomenon occurs in clinical practice and whether it may have also contributed to the low ORR in this patient population.

In ovarian cancer patients, the PFI is a useful marker of potential response to PARP inhibitors, with patients with longer PFI shown to have the most responsive tumours (Fong *et al*, 2010). The current study, unlike many of the PARP- trials in ovarian cancer, allowed recruitment of ovarian cancer patients with platinum-resistant disease (progression within 6 months of platinum-based therapy) to participate. These data confirm the findings of Fong *et al* (2010), with the greatest benefit seen in those patients with longest PFI (>12 months). Hence, inclusion of platinum resistant patients may have adversely affected the ORR.

Although dosing schedule is important for achieving clinical benefit, what is not clear is how much PARP inhibition is needed for the optimal use of these agents. In this study increasing the dose of rucaparib did not consistently result in a greater degree of PARP inhibition, as seen in Figure 3, and PK analysis failed to show any PK–PD relationship. Furthermore, the CBR was similar in the 92 mg o.d. continuous cohort (*n*=5, 80%) to the patients treated at higher and more frequent oral doses (*n*=12, 75%). Conversely, similar degrees of enzyme inhibition were seen across different dose regimens from 92 mg o.d. to 600 mg b.d. For some patients maximum PARP enzyme inhibition might be achieved at lower doses and significant further inhibition is not achieved by higher doses or even more frequent dosing. This might be important in patients unable to tolerate the higher doses of rucaparib (and other PARPis), currently under investigation in ongoing single-agent and combination studies. However, it should be remembered that in the two proof-of-concept phase 2 studies of olaparib in BRCA-mutant breast and ovarian cancer, higher rates of response were seen the patients who received the 400 mg dosing than those in the 100 mg b.d. cohort (Audeh *et al*, 2010; Tutt *et al*, 2010).

No significant difference was seen in baseline PARP activity levels between those patients who progressed after 2 cycles compared with that in patients with a CR/PR/SD ⩾12-weeks response. However, this represents a small population size and it known that baseline levels of PARP activity vary markedly in both cancer patients and healthy human volunteers (Zaremba *et al*, 2011), and may result in different clinical outcomes from the same degree of PARP inhibition.

Evaluating dose–response of rucaparib using a PD biomarker assay such as the PARP inhibition assay is challenging and has limitations. In this study, levels of PARP inhibition were assessed only in surrogate PBLs and not in tumour biopsies, and hence there may have been a disparity between inhibition levels at these sites because of drug exposure/penetration that could have affected clinical outcome. In addition, the observation of similar degrees of maximum enzyme inhibition across different doses (92 mg o.d. to 600 mg b.d.) may be because of the limitations of the PD biomarker assay and the reality may be that higher subtle differences in inhibition are seen within the tumours themselves.

The key three lessons learned from this study are: firstly, rucaparib continuous dosing to sustain PARP inhibition is needed for clinical efficacy; secondly, the recommended phase 2 dose (RP2D) for continuous oral scheduling has not been established and requires further exploration; and thirdly, the use of a PD biomarker to evaluate dose–response has its limitations.

Results and lessons learned from this study are being applied to the ongoing Clovis-sponsored studies (Study 10; NCT 01482715, ARIEL 2; NCT 01891344 and ARIEL3; NCT 01968213). Study 10 (CO-338-010) is a three-part, open-label, safety, PK and preliminary efficacy phase 1/2 study of oral rucaparib administered daily for continuous 21-day cycles. It was initiated in Q4 2011 by Clovis in an attempt to further explore the preliminary efficacy results from this study and to establish further the RP2D of oral continuous rucaparib. In part 1 (dose-escalation phase), 56 patients were enrolled (27 breast cancer, 20 ovarian/peritoneal cancer and 9 other tumours) and were treated with rucaparib at dose levels of 40, 80, 160, 300, and 500 mg once daily (o.d.) and 240, 360, 480, 600, and 840 mg b.d. Results, presented at ESMO 2014, established the RP2D of rucaparib to be 600 mg b.d. based on toxicity and preliminary efficacy data (Kristeleit *et al*, 2014). In terms of toxicity: 1 out of 6 patients treated with 360 mg b.d. experienced a DLT (grade 3 nausea despite maximal intervention); no DLTs were observed in the 480 (*n*=9), 600 (*n*=5) or 840 mg b.d. (*n*=3) cohorts during cycle 1. Overall, the most common AEs were mild to moderate GI effects and fatigue (mostly grade 1/2) and no patient discontinued rucaparib because of an AE. The study is ongoing with part 2b investigating efficacy in heavily pre-treated high-grade serous, BRCA-mutant ovarian cancers at the RP2D of 600 mg b.d. Preliminary results of ARIEL2, investigating 600 mg b.d. rucaparib, presented at ASCO 2015, reported ORR of 82% (RECIST and CA125) in patients with germline and somatic *BRCA* mutations (McNeish *et al*, 2015). In addition, interim results of study 10 part 2 have shown ORR or 77% (RECIST and CA125) in heavily pre-treated BRCA-mutant ovarian cancers (Shapira-Frommer *et al*, 2015). Developing a diagnostic signature of HRD in ovarian cancer is also a major focus of ARIEL2, with early results indicating efficacy in patients who are *BRCA* wild type but with high tumour genomic loss of heterozygosity (LOH) (McNeish *et al*, 2015).

To conclude, this study has established that rucaparib is active in gBRCA-mutant ovarian cancer, this activity correlates with PFI and that continuous rucaparib dosing is required for optimal response. The RP2D of 600 mg b.d. is now being tested in terms of tolerability and efficacy in the larger ovarian cancer population and we eagerly await the final results of these ongoing rucaparib studies.

## Figures and Tables

**Table 1:** Efficacy of rucaparib by RECIST for the 71 patients by i.v. and oral rucaparib and by BRCA mutation status and tumour type

**RECIST Response by patient no. (%)**	**All patients** ***n*****=71**	**The i.v. cohorts** ***n*****=44**	***BRCA1*** **ovary*****n*****=16**	***BRCA2*** **ovary** ***n*****=10**	***BRCA1*** **breast** ***n*****=9**	***BRCA2*** **breast** ***n*****=9**	**Oral cohorts** ***n*****=27**^a^	***BRCA1*** **ovary** ***n*****=16**	***BRCA2*** **ovary** ***n*****=3**	***BRCA1*** **breast** ***n*****=4**	***BRCA2*** **breast** ***n*****=1**	**Oral cohorts continuous dosing** ***n*****=17**
Objective response	5 (7)	1 (2)	1 (6)	0 (0)	0 (0)	0 (0)	4 (15)	3 (19)	1 (33)	0 (0)	0 (0)	3 (18)
Complete response	1 (1)	0 (0)	0 (0)	0 (0)	0 (0)	0 (0)	1 (4)	1 (6)	0 (0)	0 (0)	0 (0)	1 (6)
Partial response	4 (6)	1 (2)	1 (6)	0 (0)	0 (0)	0 (0)	3 (11)	2 (13)	1 (33)	0 (0)	0 (0)	2 (12)
Stable disease ⩾12 weeks	35 (49)	18 (41)	4 (25)	6 (60)	4 (44)	4 (44)	17 (63)	12 (75)	2 (67)	0 (0)	1 (100)	10 (59)
Progressive disease	28 (39)	22 (50)	10 (63)	3 (30)	5 (56)	4 (44)	6 (22)	1 (6)	0 (0)	4 (100)	0 (0)	4 (23)

Abbreviations: CBR=clinical benefit rate; i.v.=intravenous; RECIST=Response Evaluation Criteria in Solid Tumours.

a Includes data from three high-grade serous ovarian cancer patients who were unknown BRCA or BRCA wild type.

**Table 2:** Rucaparib-related (possibly, probably or almost certainly) adverse events (AEs) that occurred in at least 3 of all 78 patients by grade

	**Maximum CTCAE grade per patient for all dose levels, no. (%)**		
**Adverse event**	**Grade 1**	**Grade 2**	**Grade 3**
Nausea	22 (28)	4 (5)	2 (3)
Fatigue	20 (26)	15 (19)	5 (6)
Headache	12 (15)	5 (6)	0 (0)
Diarrhoea	11 (14)	1 (1)	0 (0)
Infusion site reaction^a^	5 (11)	1 (2)	0 (0)
Dizziness	8 (10)	1 (1)	0 (0)
Vomiting	7 (9)	3 (4)	0 (0)
Anorexia	7 (9)	1 (1)	0 (0)
Constipation	6 (8)	1 (1)	0 (0)
Alopecia	6 (8)	0 (0)	0 (0)
Pruritus	4 (5)	1 (1)	0 (0)
Taste alteration (dysgeusia)	4 (5)	1 (1)	0 (0)
Anaemia	0 (0)	4 (5)	0 (0)
Neutropenia	3 (4)	1 (1)	0 (0)
Dry mouth (xerostomia)	3 (4)	0 (0)	0 (0)
Elevated transaminase (AST)	3 (4)	0 (0)	0 (0)
Low mood	3 (4)	0 (0)	1 (1)
Sensory neuropathy	3 (4)	0 (0)	0 (0)
Abdominal pain	2 (3)	2 (3)	1 (1)
Arthralgia	3 (4)	2 (3)	0 (0)
Myalgia	2 (3)	1 (1)	0 (0)
Lymphopenia	1 (1)	2 (3)	0 (0)

Abbreviations: AST=aspartate transaminase; CTCAE=Common Terminology Criteria for Adverse Events.

Data are patient number (%). There were no grade 4/5 AEs.

a Intravenous (i.v.) cohort only (*n*=47).

## Notes

Nicola Curtin is an inventor on patents relating to the therapeutic use of rucaparib in combination with chemotherapy and as single agent. She has previously received research funding from Agouron for the development of rucaparib, Pfizer for preclinical studies relating to the use of rucaparib in translational studies, and Clovis Oncology for preclinical studies concerning the persistence of PARP inhibition by rucaparib. She is not currently in receipt of any financial support from these companies. Ruth Plummer is an inventor on patents relating to the use of rucaparib and has received an honorarium from Clovis Oncology in 2014. Yvette Drew has acted as an advisory board member for Clovis Oncology in 2015 and for this received an honorarium from Clovis Oncology. The remaining authors declare no conflict of interest.

## References

[bib1] Audeh MW, Carmichael J, Penson RT, Friedlander M, Powell B, Bell-McGuinn KM, Scott C, Weitzel JN, Oaknin A, Loman N, Lu K, Schmutzler RK, Matulonis U, Wickens M, Tutt A (2010) Oral poly(ADP-ribose) polymerase inhibitor olaparib in patients with BRCA1 or BRCA2 mutations and recurrent ovarian cancer: a proof-of-concept trial. Lancet 376: 245–251. 20609468 10.1016/S0140-6736(10)60893-8

[bib2] Bryant HE, Schultz N, Thomas HD, Parker KM, Flower D, Lopez E, Kyle S, Meuth M, Curtin NJ, Helleday T (2005) Specific killing of BRCA2-deficient tumours with inhibitors of poly(ADP-ribose) polymerase. Nature 434: 913–917. 15829966 10.1038/nature03443

[bib3] Chan N, Pires IM, Bencokova Z, Coackley C, Luoto KR, Bhogal N, Lakshman M, Gottipati P, Oliver FJ, Helleday T, Hammond EM, Bristow RG (2010) Contextual synthetic lethality of cancer cell kill based on the tumor microenvironment. Cancer Res 70: 8045–8054. 20924112 10.1158/0008-5472.CAN-10-2352 PMC2978949

[bib4] Drew Y, Mulligan EA, Vong WT, Thomas HD, Kahn S, Kyle S, Mukhopadhyay A, Los G, Hostomsky Z, Plummer ER, Edmondson RJ, Curtin NJ (2011a) Therapeutic potential of poly(ADP-ribose) polymerase inhibitor AG014699 in human cancers with mutated or methylated BRCA1 or BRCA2. J Natl Cancer Inst 103: 334–346. 21183737 10.1093/jnci/djq509

[bib5] Drew Y, Ledermann JA, Jones A, Hall G, Jayson GC, Highley M, Rea D, Glasspool RM, Halford SE, Crosswell G, Colebrook S, Boddy AV, Curtin NJ, Plummer ER (2011b) Phase II trial of the poly(ADP-ribose) polymerase (PARP) inhibitor AG-014699 in BRCA 1 and 2–mutated, advanced ovarian and/or locally advanced or metastatic breast cancer. J Clin Oncol 29(3104).

[bib6] Farmer H, McCabe N, Lord CJ, Tutt ANJ, Johnson DA, Richardson TB, Santarosa M, Dillon KJ, Hickson I, Knights C, Martin NMB, Jackson SP, Smith GCM, Ashworth A (2005) Targeting the DNA repair defect in BRCA mutant cells as a therapeutic strategy. Nature 434: 917–921. 15829967 10.1038/nature03445

[bib7] Fong PC, Boss DS, Yap TA, Tutt A, Wu P, Mergui-Roelvink M, Mortimer P, Swaisland H, Lau A, O'Connor MJ, Ashworth A, Carmichael J, Kaye SB, Schellens JH, de Bono JS (2009) Inhibition of poly(ADP-ribose) polymerase in tumors from BRCA mutation carriers. N Engl J Med 361: 123–134. 19553641 10.1056/NEJMoa0900212

[bib8] Fong PC, Yap TA, Boss DS, Carden CP, Mergui-Roelvink M, Gourley C, De Greve J, Lubinski J, Shanley S, Messiou C, A'Hern R, Tutt A, Ashworth A, Stone J, Carmichael J, Schellens JH, de Bono JS, Kaye SB (2010) Poly(ADP)-ribose polymerase inhibition: frequent durable responses in BRCA carrier ovarian cancer correlating with platinum-free interval. J Clin Oncol 28: 2512–2519. 20406929 10.1200/JCO.2009.26.9589

[bib9] Gelmon KA, Tischkowitz M, Mackay H, Swenerton K, Robidoux A, Tonkin K, Hirte H, Huntsman D, Clemons M, Gilks B, Yerushalmi R, Macpherson E, Carmichael J, Oza A (2011) Olaparib in patients with recurrent high-grade serous or poorly differentiated ovarian carcinoma or triple-negative breast cancer: a phase 2, multicentre, open-label, non-randomised study. Lancet Oncol 12: 852–861. 21862407 10.1016/S1470-2045(11)70214-5

[bib10] Gudmundsdottir K, Ashworth A (2006) The roles of BRCA1 and BRCA2 and associated proteins in the maintenance of genomic stability. Oncogene 25: 5864–5874. 16998501 10.1038/sj.onc.1209874

[bib11] Helleday T, Lo J, van Gent DC, Engelward BP (2007) DNA double-strand break repair: from mechanistic understanding to cancer treatment. DNA Repair (Amst) 6: 923–935. 17363343 10.1016/j.dnarep.2007.02.006

[bib12] Kaelin WG (2005) The concept of synthetic lethality in the context of anticancer therapy. Nat Rev Cancer 5: 689–698. 16110319 10.1038/nrc1691

[bib13] Kristeleit R, Shapira-Frommer R, Burris H, Patel MR, Lorusso PM, Oza AM, Balmaña J, Domchek SM, Chen L, Montes A, Plummer R, Arkenau H, Maloney L, Dominy E, Shapiro G (2014) Phase 1/2 study of oral rucaparib: updated phase 1 and preliminary phase 2 results. ESMO Ann Oncol 25(Suppl 4): iv305–iv326.

[bib14] Liu JF, Barry WT, Birrer M, Lee JM, Buckanovich RJ, Fleming GF, Rimel B, Buss MK, Nattam S, Hurteau J, Luo W, Quy P, Whalen C, Obermayer L, Lee H, Winer EP, Kohn EC, Ivy SP, Matulonis UA (2014) Combination cediranib and olaparib versus olaparib alone for women with recurrent platinum-sensitive ovarian cancer: a randomised phase 2 study. Lancet Oncol 15: 1207–1214. 25218906 10.1016/S1470-2045(14)70391-2 PMC4294183

[bib15] McNeish IA, Oza AM, Coleman RL, Scott CL, Konecny GE, Tinker A, O'Malley DM, Brenton J, Kristeleit RS, Bell-McGuinn K, Oaknin A, Leary A, Lin K, Raponi M, Giordano H, Goble S, Rolfe L, Yelensky R, Allen AR, Swisher EM (2015) Results of ARIEL2: a phase 2 trial to prospectively identify ovarian cancer patients likely to respond to rucaparib using tumor genetic analysis. J Clin Oncol (Meeting Abstracts) 33(suppl): abstract 5508.

[bib16] Molife LR, Roxburgh P, Wilson RH, Gupta A, Middleton MR, Jeffry Evans TR, Michie C, Mateo J, Crawford D, Eatock M, Saka W, Cresti N, Drew Y, Giordano H, Despain D, Simpson D, Allen A, Jaw-Tsai S, Plummer R (2013) A phase I study of oral rucaparib in combination with carboplatin. J Clin Oncol 31(suppl): abstract 2586).

[bib17] Mukhopadhyay A, Elattar A, Cerbinskaite A, Wilkinson SJ, Drew Y, Kyle S, Los G, Hostomsky Z, Edmondson RJ, Curtin NJ (2010) Development of a functional assay for homologous recombination status in primary cultures of epithelial ovarian tumor and correlation with sensitivity to poly(ADP-ribose) polymerase inhibitors. Clin Cancer Res 16: 2344–2351. 20371688 10.1158/1078-0432.CCR-09-2758

[bib18] Murray J, Thomas H, Berry P, Kyle S, Patterson M, Jones C, Los G, Hostomsky Z, Plummer ER, Boddy AV, Curtin NJ (2014) Tumour cell retention of rucaparib, sustained PARP inhibition and efficacy of weekly as well as daily schedules. Br J Cancer 110: 1977–1984. 24556618 10.1038/bjc.2014.91 PMC3992512

[bib19] Plummer ER, Middleton MR, Jones C, Olsen A, Hickson I, McHugh P, Margison GP, McGown G, Thorncroft M, Watson AJ, Boddy AV, Calvert AH, Harris AL, Newell DR, Curtin NJ (2005) Temozolomide pharmacodynamics in patients with metastatic melanoma: DNA damage and activity of repair enzymes 6-alkylguanine alkyltransferase and poly(ADP-ribose) polymerase-1. Clin Cancer Res 11: 3402–3409. 15867241 10.1158/1078-0432.CCR-04-2353

[bib20] Plummer R, Jones C, Middleton M, Wilson R, Evans J, Olsen A, Curtin N, Boddy A, McHugh P, Newell D, Harris A, Johnson P, Steinfeldt H, Dewji R, Wang D, Robson L, Calvert H (2008) Phase I study of the poly (ADP-ribose) polymerase inhibitor, AG014699, in combination with temozolomide in patients with advanced solid tumors. Clin Cancer Res 14: 7917–7923. 19047122 10.1158/1078-0432.CCR-08-1223 PMC2652879

[bib21] Plummer R, Lorigan P, Steven N, Scott L, Middleton MR, Wilson RH, Mulligan E, Curtin N, Wang D, Dewji R, Abbattista A, Gallo J, Calvert H (2013) A phase II study of the potent PARP inhibitor, Rucaparib (PF-01367338, AG014699), with temozolomide in patients with metastatic melanoma demonstrating evidence of chemopotentiation. Cancer Chemother Pharmacol 71: 1191–1199. 23423489 10.1007/s00280-013-2113-1

[bib22] Sandhu SK, Schelman WR, Wilding G, Moreno V, Baird RD, Miranda S, Hylands L, Riisnaes R, Forster M, Omlin A, Kreischer N, Thway K, Gevensleben H, Sun L, Loughney J, Chatterjee M, Toniatti C, Carpenter CL, Iannone R, Kaye SB, de Bono JS, Wenham RM (2013) The poly (ADP-ribose) polymerase inhibitor niraparib (MK4827) in BRCA mutation carriers and patients with sporadic cancer: a phase 1 dose-escalation trial. Lancet Oncol 14: 882–892. 23810788 10.1016/S1470-2045(13)70240-7

[bib23] Schreiber V, Dantzer F, Ame JC, de Murcia G (2006) Poly(ADP-ribose): novel functions for an old molecule. Nat Rev Mol Cell Biol 7: 517–528. 16829982 10.1038/nrm1963

[bib24] Shapira-Frommer R, Oza AM, Domchek SM, Balmaña J, Patel MR, Chen LM, Drew Y, Burris HA III, Korach J, Flynn M, Bowering VL, Morgan MA, Watkins SP, Simpson D, Goble S, Maloney L, Kristeleit RS (2015) A phase 2 open-label, multicenter study of single-agent rucaparib in the treatment of patients with relapsed ovarian cancer and a deleterious BRCA mutation. Ann Oncol 33, (suppl): abstract 2746.

[bib25] Sonnenblick A, de Azambuja E, Azim HA Jr, Piccart M (2015) An update on PARP inhibitors—moving to the adjuvant setting. Nat Rev Clin Oncol 1: 27–41. 10.1038/nrclinonc.2014.163 25286972

[bib26] Swisher EM, Sakai W, Karlan BY, Wurz K, Urban N, Taniguchi T (2008) Secondary BRCA1 mutations in BRCA1-mutated ovarian carcinomas with platinum resistance. Cancer Res 68: 2581–2586. 18413725 10.1158/0008-5472.CAN-08-0088 PMC2674369

[bib27] The Cancer Genome Atlas Research Network (2011) Integrated genomic analyses of ovarian carcinoma. Nature 474: 609–615. 21720365 10.1038/nature10166 PMC3163504

[bib28] Therasse P, Arbuck SG, Eisenhauer EA, Wanders J, Kaplan RS, Rubinstein L, Verweij J, Van Glabbeke M, van Oosterom AT, Christian MC, Gwyther SG (2000) New guidelines to evaluate the response to treatment in solid tumors. European Organization for Research and Treatment of Cancer, National Cancer Institute of the United States, National Cancer Institute of Canada. J Natl Cancer Inst 92: 205–216. 10655437 10.1093/jnci/92.3.205

[bib29] Thomas HD, Calabrese CR, Batey MA, Canan S, Hostomsky Z, Kyle S, Maegley KA, Newell DR, Skalitzky D, Wang LZ, Webber SE, Curtin NJ (2007) Preclinical selection of a novel poly(ADP-ribose) polymerase inhibitor for clinical trial. Mol Cancer Ther 6: 945–956. 17363489 10.1158/1535-7163.MCT-06-0552

[bib30] Tutt A, Robson M, Garber JE, Domchek SM, Audeh MW, Weitzel JN, Friedlander M, Arun B, Loman N, Schmutzler RK, Wardley A, Mitchell G, Earl H, Wickens M, Carmichael J (2010) Oral poly(ADP-ribose) polymerase inhibitor olaparib in patients with BRCA1 or BRCA2 mutations and advanced breast cancer: a proof-of-concept trial. Lancet 376: 235–244. 20609467 10.1016/S0140-6736(10)60892-6

[bib31] Wahlberg E, Karlberg T, Kouznetsova E, Markova N, Macchiarulo A, Thorsell AG, Pol E, Frostell Å, Ekblad T, Öncü D, Kull B, Robertson GM, Pellicciari R, Schüler H, Weigelt J (2012) Family-wide chemical profiling and structural analysis of PARP and tankyrase inhibitors. Nat Biotechnol 30: 283–288. 22343925 10.1038/nbt.2121

[bib32] Zaremba T, Thomas HD, Cole M, Coulthard SA, Plummer ER, Curtin NJ (2011) Poly(ADP-ribose) polymerase-1 (PARP-1) pharmacogenetics, activity and expression analysis in cancer patients and healthy volunteers. Biochem J 436: 671–679. 21434873 10.1042/BJ20101723

