# Phase 2 multicentre trial investigating intermittent and continuous dosing schedules of the poly(ADP-ribose) polymerase inhibitor rucaparib in germline BRCA mutation carriers with advanced ovarian and breast cancer

**DOI:** 10.1038/bjc.2016.133

**Published:** 2016-05-26

**Authors:** Yvette Drew, Jonathan Ledermann, Geoff Hall, Daniel Rea, Ros Glasspool, Martin Highley, Gordon Jayson, Julieann Sludden, James Murray, David Jamieson, Sarah Halford, Gary Acton, Zoe Backholer, Raffaella Mangano, Alan Boddy, Nicola Curtin, Ruth Plummer

**Correction to:** *British Journal of Cancer* (2016) **114**, 723–730; doi:10.1038/bjc.2016.41; published online 22 March 2016

**Updated online 26 May 2016:** This article was originally published under a standard BJC licence, but has now been made available under a CC BY 4.0 licence. The PDF and HTML versions of the paper have been modified accordingly.

